# Successful endoscopic closure of a duodenal anastomotic leak using a through-the-scope suturing device

**DOI:** 10.1055/a-2936-0301

**Published:** 2026-09-10

**Authors:** Lidia Marti Romero, Vanesa Martinez Escapa, Tatiana Barberá Martinez, Carmen Pla Cortina, Miguel Oviedo Bravo

**Affiliations:** 1 Digestive section 16801 Hospital Arnau de VilanovaValenciaValencian CommunitySpain; 2 Anesthesia Department 16801 Hospital Arnau de VilanovaValenciaValencian CommunitySpain; 3 General Surgery Department 16801 Hospital Arnau de VilanovaValenciaValencian CommunitySpain

The Endoscopic HeliX Tacking System is a through-the-scope suture-based device approved for tissue approximation of mucosal defects in the upper and lower gastrointestinal tract. 1​ ​2​ Although primarily indicated for closing mucosal defects, in this video ( Video 1 ), we expanded its clinical applications to address a more complex scenario: the successful closure of a duodenal anastomosis leak. 3​

**Video 1** The Helix tacking system. Video URI:


A 68-year-old man underwent laparoscopic resection of the third and fourth portions of the duodenum, with a side-to-side duodenojejunal anastomosis for the treatment of a duodenal gastrointestinal stromal tumor. Forty-eight hours after surgery, the patient presented abdominal pain, fever, and elevated inflammatory laboratory parameters. A computed tomographic scan confirmed an anastomotic leak. Due to the lack of clinical improvement despite antibiotic therapy and parenteral nutrition, percutaneous drainage of the collection and endoscopic treatment were indicated.

**Fig. 1:**
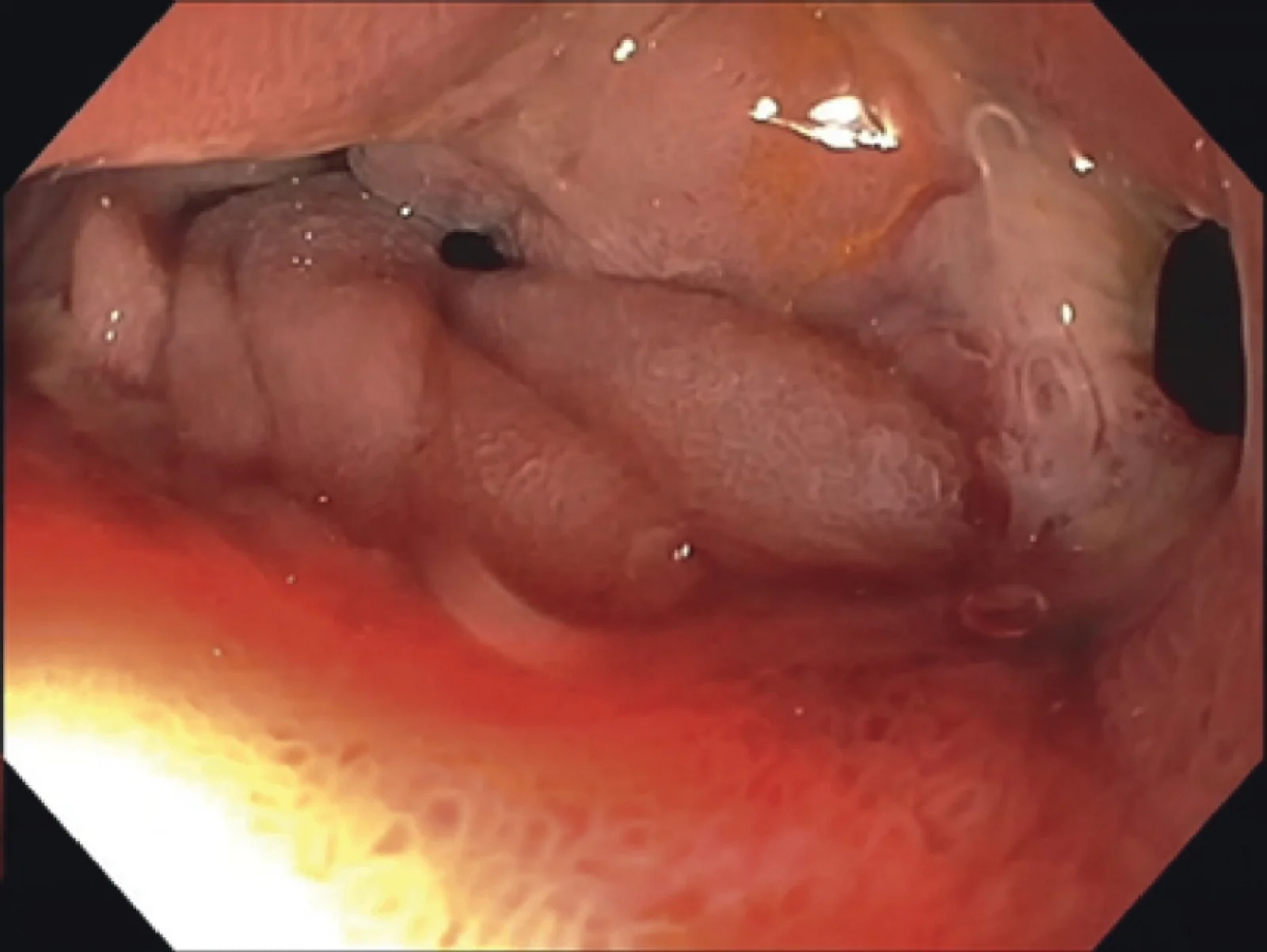
An anastomotic leak.

**Fig. 2:**
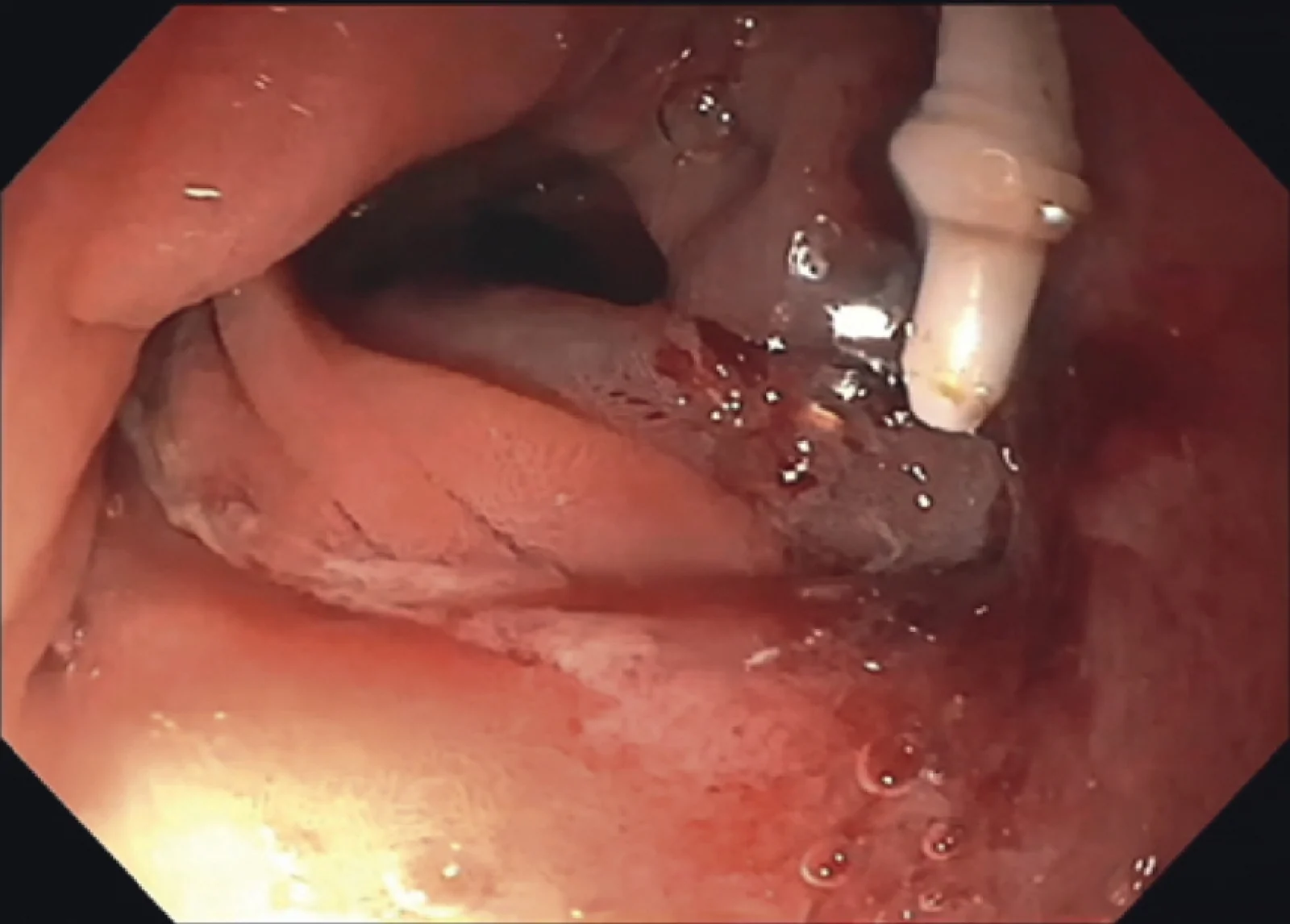
Helix closure.

Gastroscopy performed 7 days after surgery revealed a 10 mm suture dehiscence ( Fig. 1 ). The defect was closed using an endoscopic suturing device (HeliX Tacking System) with four tacks. Argon was applied on the edges; we placed the four tacks following an x pattern and tension is applied before cutting the suture ( Fig. 2 ). Successful closure was confirmed with fluoroscopy, which showed no contrast extravasation following intraluminal injection.

**Fig. 3:**
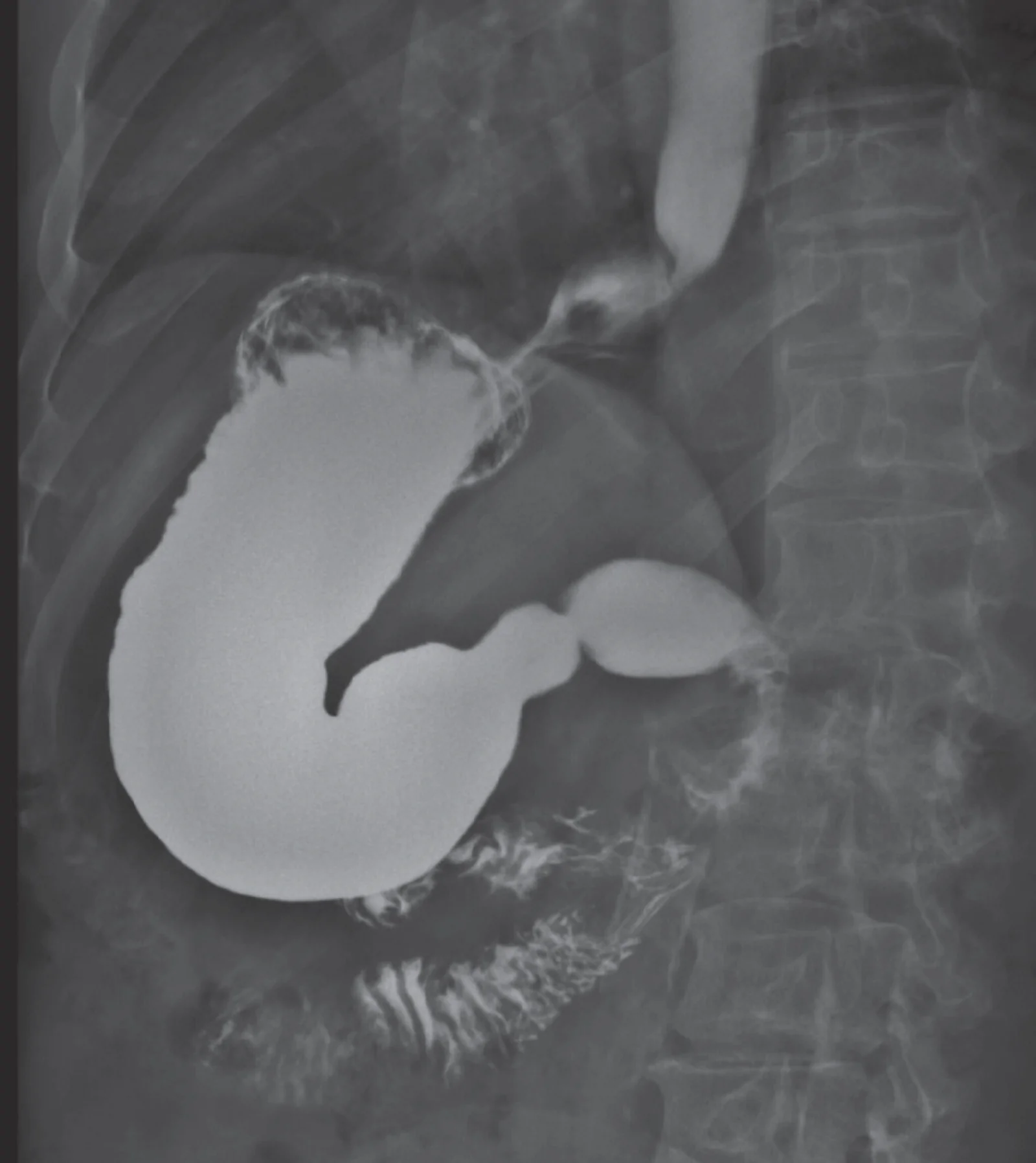
GI transit.

The patient’s clinical course was favorable, showing a progressive decrease in inflammatory parameters. Radiological follow-up confirmed the resolution of the leak, allowing for the resumption of oral intake. A follow-up gastroscopy performed 1 week later showed an intact endoscopic suture with no contrast extravasation and a patent intestinal limb. At the 2-month follow-up, the patient remained asymptomatic, and a small bowel series revealed no evidence of contrast leakage or stenosis ( Fig. 3 ).

The management of complex gastrointestinal defects (CGDs), such as fistulas, leaks, and anastomotic dehiscence, remains challenging. The through-the-scope suturing system offers a minimally invasive alternative for CGD closure without requiring scope withdrawal. 4​

Endoscopy_UCTN_Code_TTT_1AO_2AI Endoscopy_UCTN_Code_TTT_1AO_2A
